# Recent advances in the biology and drug targeting of malaria parasite aminoacyl-tRNA synthetases

**DOI:** 10.1186/s12936-016-1247-0

**Published:** 2016-04-12

**Authors:** Sameena Khan

**Affiliations:** Drug Discovery Research Centre, Translational Health Science and Technology Institute (THSTI), NCR Biotech Science Cluster, 3rd Milestone, Faridabad-Gurgaon Expressway, PO box #04, Faridabad, 121001 India

## Abstract

Escalating drug resistance in malaria parasites and lack of vaccine entails the discovery of novel drug targets and inhibitor molecules. The multi-component protein translation machinery is a rich source of such drug targets. Malaria parasites contain three translational compartments: the cytoplasm, apicoplast and mitochondrion, of which the latter two are of the prokaryotic type. Recent explorations by many groups into the malaria parasite protein translation enzymes, aminoacyl-tRNA synthetases (aaRSs), have yielded many promising inhibitors. The understanding of the biology of this unique set of 36 enzymes has become much clearer in recent times. Current review discusses the advances made in understanding of crucial aaRSs from *Plasmodium* and also the specific inhibitors found against malaria aaRSs.

## Background

*Plasmodium falciparum* causes the most lethal form of malaria and is the world’s largest killer with ~ 438,000 deaths and more than 200 million infections annually [1]. While the 2015 Nobel prize in physiology celebrates the triumph over deadly malarial and worm parasites, drug resistance among pathogens of bacterial and eukaryotic origin, including malaria parasites and worms is inevitable. The current situation is worsened by the increasing drug resistance in malaria parasites, even to mainstream drugs in clinical use, such as artemisinins [1]. Vaccination programmes have not been successful yet, which makes it urgent to find new molecular scaffolds to design efficient anti-malarials [1]. The highly complex progression of the parasite through its life cycle depends on its varying its proteome to fit different cellular milieus of vector salivary gland, gut, human blood stream, hepatocytes and erythrocytes [2–4]. A dynamic proteome presents problems for selecting multistage targets as reflected in the inefficacy of many drugs in clinical use on the liver stage. In this direction, housekeeping pathways, such as protein translation, are attractive drug targets as they are not only vital but also active in all stages [5].

**Fig. 1:**
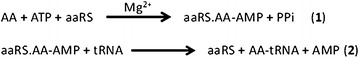
Generalized two step aminoacylation reaction. In the first step, specific amino acid (AA) is combined with ATP molecule to form a tightly bound aminoacyl-adenylate complex (AA-AMP) by release of pyrophosphate (PPi) and help of a divalent cation. In second step, the activated amino acid is transferred to the 3′ end of cognate tRNA molecule to form charged tRNA (AA-tRNA) with release of AMP. These charged tRNA molecules are then used by ribosomes for protein translation

The malaria parasite contains three genomes; nuclear, apicoplastic (a relic chloroplast) and mitochondrial and all three genomes require dedicated translational machineries to function [5]. Protein translation machinery provides a diverse collection of proteins to be targeted and malarial aminoacyl-tRNA synthetases (aaRSs) have received the most attention for drug targeting in the last half-decade [5, 6]. aaRSs catalyze the first reaction of protein biosynthesis by combining a specific amino acid to cognate tRNA molecules in a two-step reaction (Fig. 1) [7]. Generally, there are 20 different aminoacyl-tRNA synthetases in a protein translational compartment, specific to one of the twenty amino acids [7–9]. Depending on the architecture of the active site and mode of tRNA binding, aaRSs are divided into two structural classes, with 10 enzymes in each class [7–9]. aaRSs are one of the most ancient enzymes and over the course of evolution, have appended additional domains to their core structure to perform additional non-canonical functions [10, 11]. These functional expansions range from splicing, cytokine-like function to roles in DNA damage response. Molecular details, structures and a fundamental understanding of workings of aaRSs, including their moonlighting functions, are available in great detail and discussed in many reviews [7–11].

Protein translation ensures a high fidelity by quality checks at several steps [12, 13]. Proofreading at the aminoacylation step to discriminate between cognate amino acid and isosteric substrates is performed by an editing pocket appended (*cis*) to many aaRSs and by *trans*-editing factors [12–14]. Class I enzymes contain an insertion in their Rossmann fold called *connective polypeptide 1* (CP1), which in some cases forms the editing pocket [14]. CP1 can catalyze the reversion of both pre- and post-transferred errors in aminoacylation. Class II aaRSs contain a distinct editing domain, which mostly hydrolyse the mischarged tRNAs (post-transfer). *Trans*-editing factors like AlaX and Ybak hydrolyse misacylated tRNAs [12–14]. Enantiomeric selectivity is provided by the D-tyrosyl-tRNA deacylase (DTD) enzyme, which hydrolyses D-amino acids coupled to tRNA molecules [5, 15, 16].

## Reduced set of aaRSs translate parasite genome efficiently

**Fig. 2:**
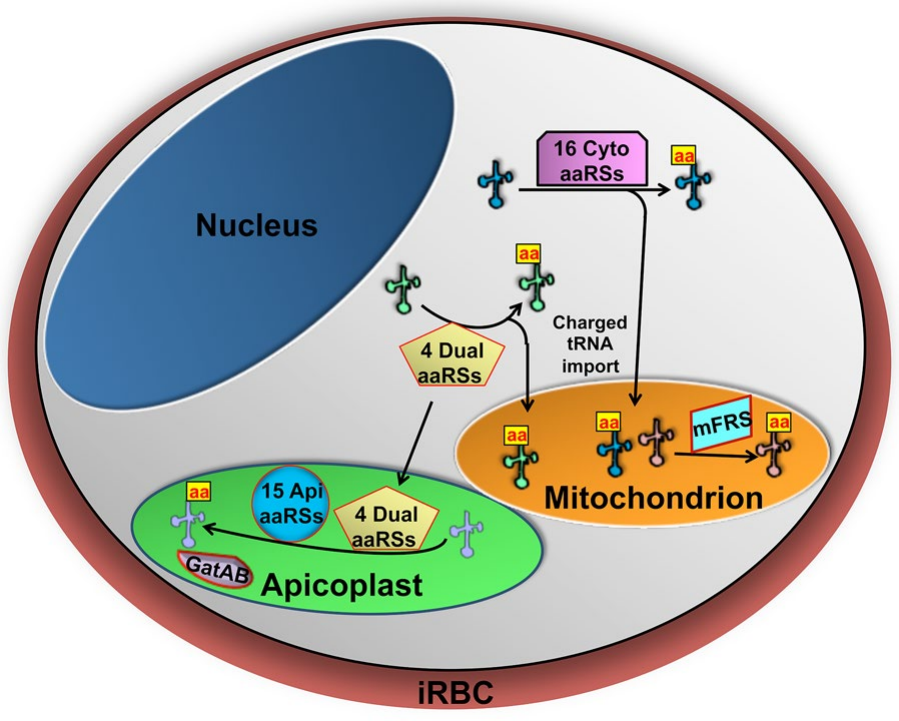
Cellular distribution of 36 malaria parasite aminoacyl-tRNA synthetases (aaRSs). All 36 aaRSs are encoded by the nuclear genome. 16 aaRSs are exclusively present in cytoplasm (Cyto aaRSs) of parasite and 15 are exclusive to the apicoplast (Api aaRSs). Four aaRSs; AlaRS, ThrRS, GlyRS and CysRS are shared by both apicoplast and the cytoplasm by mechanism of dual localization (denoted as Dual aaRSs). A unique amidotransferase (GatAB) provides the glutamine charged cognate tRNA in the apicoplast. Mitochondrion contain only one enzymatically active aaRS; PheRS (mFRS). Mitochondrion seem to be reliant on the charged tRNA import from the cytoplasm for its translation. tRNAs charged with amino acid are shown with aa written in *yellow box*

Research on crucial malaria parasite aaRSs was majorly initiated with their genomic analysis and tabulation in 2008 by Bhatt et al. [17]. Their comprehensive analysis revealed that malaria parasite *P. falciparum* contains 37 aaRS genes in its nucleus, which can form 36 enzymes [17] (Table 1). Many interesting aspects about malaria aaRSs came to light through this study. For instance, compared to other organisms, malarial aaRSs constitute a much larger fraction of the overall proteome. Additionally, these aaRSs have an unusual domain architecture and contain additional domains [17]. Most intriguingly, it was, till recently, unclear how 36 aaRSs, instead of the theoretically required 60 aaRSs, provide charged tRNAs to three translational compartments; cytoplasm, mitochondrion and apicoplast (20 tRNAs per compartment being the theoretical requirement). Studies mainly focused on cellular distribution of aaRSs and import of cytoplasmically charged tRNA to mitochondrion have now revealed the scheme by which the malaria parasite efficiently utilizes a compromised array of 36 aaRSs to synthesize its proteome (Table 1) (Fig. 2). Localization studies combined with robust bioinformatics predictions have revealed that there are 16 aaRSs exclusive to cytoplasm and 15 nucleus-encoded aaRSs exclusively targeted to apicoplast (Table 1) [17–26]. Four single copy aaRSs (alanyl-tRNA synthetase; AlaRS, threonyl-tRNA synthetase; ThrRS, cysteinyl-tRNA synthetase; CysRS and glycyl-tRNA synthetase; GlyRS) are shared between the apicoplast and cytoplasm by dual localizations, where mechanisms like alternative splicing (CysRS) and presumably, alternative translation initiation (AlaRS, ThrRS and GlyRS) occur (Table 1) (Fig. 2) [18–20]. Moreover, since the apicoplast lacks glutaminyl-tRNA synthetase (GlnRS), a charged glutamine-specific tRNA is provided by the reactions of two apicoplastic enzymes; glutamyl-tRNA synthetase (GluRS) and a unique glutamyl-tRNA amidotransferase (GatAB) [27, 28]. Apicoplastic non-discriminating GluRS mischarges glutamine-specific tRNA with glutamic acid followed by tRNA-bound glutamic acid conversion into glutamine by the heterodimeric GatAB, thus providing a complete set of 20 charged tRNAs (Fig. 2) [27, 28].

**Table 1:** Genes encoding *P. falciparum* aaRSs and their localization

Protein name	Mitochondria	Apicoplast	Cytoplasm
Class I			
Arginyl-tRNA synthetase		PF3D7_0913900	PF3D7_1218600
Cysteinyl-tRNA synthetase		PF3D7_1015200.1	PF3D7_1015200.1
Glutamyl-tRNA synthetase		PF3D7_1357200	PF3D7_1349200
Glutaminyl-tRNA synthetase			PF3D7_1331700
Isoleucyl-tRNA synthetase		PF3D7_1225100^a^	PF3D7_1332900^a^
Leucyl-tRNA synthetase		PF3D7_0828200^a^	PF3D7_0622800
Methionyl-tRNA synthetase		PF3D7_1005000	PF3D7_1034900
Tryptophanyl-tRNA synthetase		PF3D7_1251700^a^	PF3D7_1336900
Tyrosyl-tRNA synthetase		PF3D7_1117500	PF3D7_0807900
Valyl-tRNA synthetase		PF3D7_0311200^a^	PF3D7_1461900
Class II			
Alanyl-tRNA synthetase		PF3D7_1367700^a^	PF3D7_1367700^a^
Asparaginyl-tRNA synthetase		PF3D7_0509600	PF3D7_0211800
Aspartyl-tRNA synthetase		PF3D7_0514300	PF3D7_0102900
Glycyl-tRNA synthetase		PF3D7_1420400	PF3D7_1420400
Histidyl-tRNA synthetase		PF3D7_0934000	PF3D7_1445100
Lysyl-tRNA synthetase		PF3D7_1416800	PF3D7_1350100
Phenylalanyl-tRNA synthetase	PF3D7_0603700	PF3D7_1232000	PF3D7_1104000 (α) PF3D7_0109800^a^ (β)
Prolyl-tRNA synthase		PF3D7_0925300^a^	PF3D7_1213800
Seryl-tRNA synthetase		PF3D7_1216000	PF3D7_071770
Threonyl-tRNA synthetase		PF3D7_1126000^a^	PF3D7_1126000^a^
Other enzymes			
D tyrosyl-tRNA deacylase			PF3D7_1108200^a^
P43			PF3D7_1442300
Glutamyl-tRNA amidotransferase		PF3D7_0416100 (A) PF3D7_0628800 (B)	

^a^ Indicate genes containing editing activity

*Plasmodium falciparum* mitochondrion was shown to harbour an enzymatically active mitochondrial phenylalanyl-tRNA synthetase (PheRS), which is unique to Plasmodium as it is absent in other apicomplexans [29]. Mitochondrial PheRS is the only aaRS present in parasite mitochondrion and its functional relevance remains unclear. The mitochondrion seem to be dependent on charged tRNA import for synthesizing its three respiratory chain associated genes; cytochrome c oxidase subunits I and III (COX1, COX3) and cytochrome b (Cytb) (Fig. 2) [5, 29, 30]. Recently, evidence for import of cytoplasmically charged phenylalanine and cysteine tRNAs was provided which suggest that the same is likely true for other tRNAs [29]. Similar studies on Toxoplasma have demonstrated the presence of an analogous translational setup in mitochondrion [31].

While the aminoacylation requirements of three translationally active compartments in *P. falciparum* are the same, it was shown that proofreading requirements at the aminoacylation level are not the same for apicoplast and cytoplasm [18]. The apicoplast seem to be tolerant for mischarged tRNAs as it only contains three aaRSs with editing pocket (Table 1) (Fig. 2). The same would not be true for the mitochondrion as it was shown that parasite mitochondrion import charged tRNAs from cytoplasm and hence fidelity would be similar to cytoplasm [29].

aaRSs can also form a highly efficient aminoacylation ensemble called a multi-synthetase complex, which consists of nine aaRSs tethered by scaffold proteins such as P43, P18 and P38 in higher eukaryotes [10]. Bioinformatics analysis of malarial aaRSs identified only one putative cytoplasmic adaptor protein, P43, that could participate in the formation of the multi-synthetase complex [17]. Plasmodium-related apicomplexan *Toxoplasma gondii* possesses a reduced multi-synthetase complex consisting of P43, methionyl- (MetRS), glutaminyl-, glutamyl-, and tyrosyl- (TyrRS) tRNA synthetases [32]. A similar reduced P43-dependent complex can be expected for malaria parasite.

## Non-canonical functions by malaria parasite aaRSs

aaRSs have not been comprehensively studied for their non-canonical functions in malaria parasite though studies suggest that malaria parasite aaRSs have evolved to meet parasite-specific needs [17–26, 33].

### Tyrosyl-tRNA synthetase modulates host immune response

**Fig. 3:**
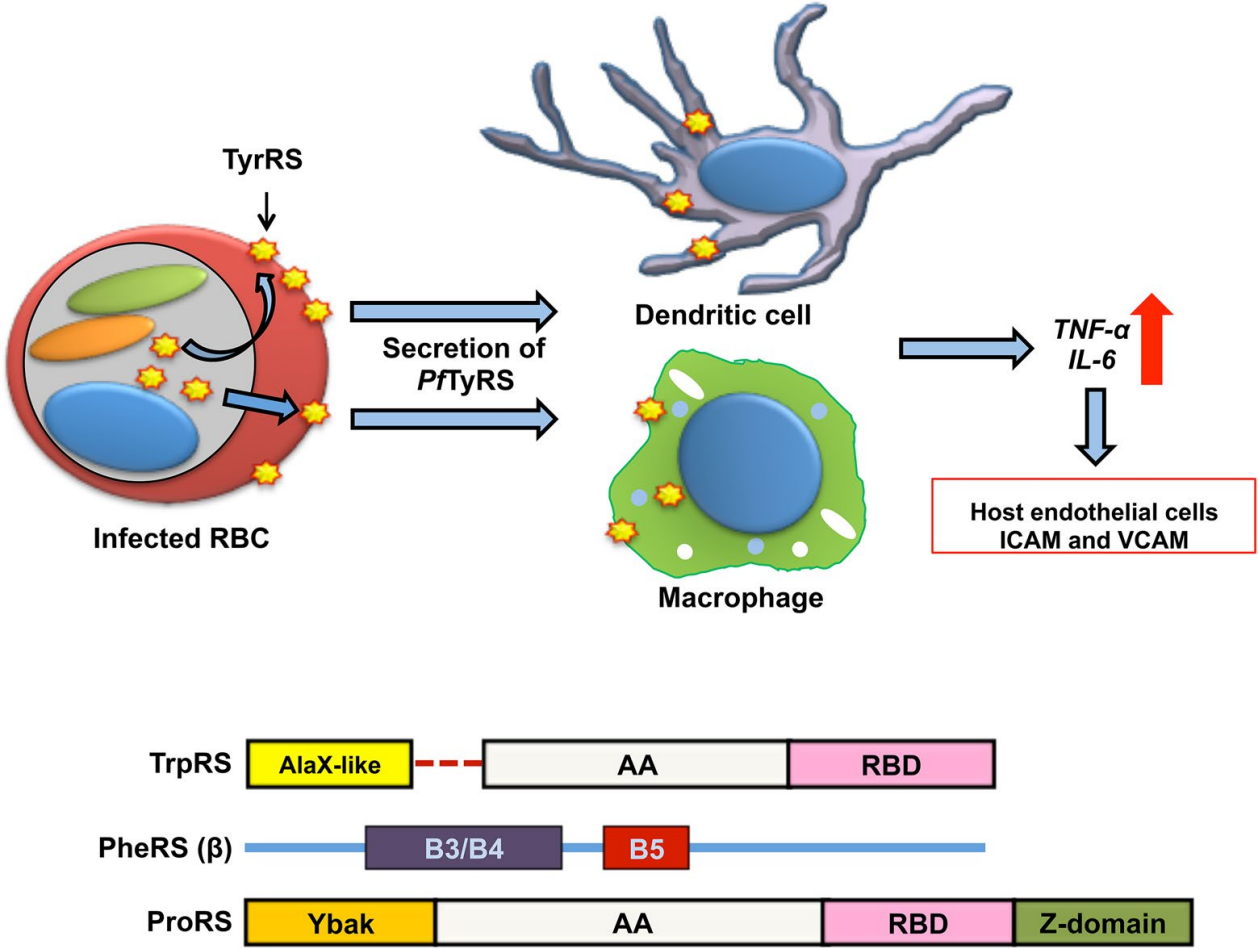
Additional functionalities and domains in *Plasmodium falciparum* aaRSs. *Plasmodium falciparum* TyrRS (shown as *yellow rhombus*) contain the ELR motif that helps it act as a cytokine to modulate immune functioning. *Pf* TyrRS is secreted during schizont burst from the infected red blood cells into blood stream. Released TyrRS interacts with dendritic cells and macrophages and gets internalized. This triggers release of TNF and IL6 and consequently results in increased host endothelium cell ICAM and VCAM expression. *Lower panel* shows aaRSs containing additional domain. N-terminal AlaX domain is present in the TrpRS and required for tRNA binding. β subunit of cytoplasmic PheRS contains a DNA binding domain B5. Cytoplasmic ProRS contain homologue of trans-editing factor Ybak fused to its N-terminal. AA, RBD and B3/B4 represent the aminoacylation domain, tRNA anticodon binding domain and the editing domain, respectively

Most prominent example of parasite specific adaptation and non-canonical functionality is the *P. falciparum* cytoplasmic *Pf*TyrRS, which can modulate host immune response [23]. Human TyrRS contains a C-terminal endothelial monocyte-activating polypeptide II (EMAPII) domain and a tripeptide cytokine motif (ELR; Glu-Leu-Arg) embedded in its catalytic domain (Rossmann fold) [10, 34]. Cytokine activities of both EMAPII and ELR are well studied [10, 34]. After cleaving into two fragments, the C-terminal fragment (EMAPII) performs cytokine-like functions such as inflammation and the N-terminal performs functions similar to interleukin 8—like cytokines such as angiogenesis [10, 34]. Malaria parasite TyrRS lacks the C-terminal EMAPII domain, but possesses the ELR motif [23]. This enzyme was observed to be present on RBC membrane in the infected RBCs and secreted outside upon schizont burst (Fig. 3) [23]. This secreted TyrRS is capable of eliciting immune modulation by binding to macrophages and dendritic cells using ELR motif and triggering secretion of pro-inflammatory cytokines TNF and IL6 (Fig. 3). Structural data showed that the *Pf*TyrRS ELR motif, unlike its human counterpart, is not buried but instead is exposed. Thus the enzyme without cleavage can probably, upon secretion, bind CXCR2 receptors present on macrophages and dendritic cells (Fig. 3) [23].

### Lysyl-tRNA synthetase can synthesize signaling molecule Ap4A

Another example is lysyl-RNA synthetase (LysRS) that can synthesize signaling molecules Ap4A (diadenosine tetraphosphate) and Ap5A (diadenosine pentaphosphate) which can regulate variety of cellular functions ranging from gene transcription, apoptosis and DNA replication to ion channel regulation [10, 35]. Malaria parasite LysRS is also capable of synthesizing an Ap4A molecule and presence of an Ap4A hydrolase in the parasite hints at a special role for this molecule in parasite physiology [25, 36]. Regulations of Ap4A synthesis and its functional relevance to parasite physiology have not yet been studied.

### Tryptophanyl-tRNA synthetase has an unusual architecture

Another unusual aaRS that malaria parasite possesses is tryptophanyl-tRNA synthetase (TrpRS) which contain a *trans*-editing factor AlaX fused to its N-terminal (Fig. 3) [17, 18, 26]. AlaX was found to be essential for functioning of the enzyme, while the enzyme without this domain was non-functional [26]. It was suggested that AlaX could assist tRNA binding to TrpRS. This is a unique feature absent in the human counterpart or any other reported mammalian TrpRS. Bioinformatics and modelling studies on TrpRS suggest that it has lost the crucial residues for editing function when compared with the *Pyrococcus horikoshii* AlaX. [26]. Human TrpRS is secreted outside cells and is an angiostatic cytokine [10, 11]. The plasmodial enzyme was found to be non-secretory in the asexual blood stages of parasite [26]. TrpRSs in human and other organisms are also capable of synthesizing signaling molecule Ap3A, but whether the malarial enzyme can synthesize the same has not been tested [10].

### Other aaRSs with extra domains in malaria parasite

Many other aaRSs were found with unusual domains in *P. falciparum* and experimental validation of their functionalities remains to be performed. For example, cytoplasmic PheRS contains DNA binding domains in its β subunit [11, 17, 29]. PheRSs have been suggested to bind to DNA and their role on DNA binding is not clear (Fig. 3) [10, 11, 29]. Cytoplasmic PheRS was observed only in the cytoplasm of parasite in all asexual blood stages and gametocyte stages of parasite suggesting that the enzyme either goes to nucleus in other stages of the life cycle (hepatocyte or mosquito) or has a conditional nuclear localization [29].

Glutathione-S-transferase (GST) or GST-like domains have important implications in protein–protein interactions such as formation of the multi-synthetase complex [10, 11, 23]. MetRS and GlnRS from *P. falciparum* were found to contain GST domains [17, 24]. Functions of these GST domains in malaria parasite aaRSs remain unclear [24]. Plasmodium GST-like domain appended to the cytoplasmic *Pf*MetRS differs from the orthologous group suggesting different functionality in different members.

Malarial prolyl-tRNA synthetase (ProRS) contain an N-terminal Ybak domain, which can potentially hydrolyze the aminoacylation bond on proline tRNA mischarged with alanine or cysteine (Fig. 3) [17, 18, 22]. The C-terminal part of *Pf*ProRS contains a pseudo-zinc binding domain, which is functional in the human counterpart [18, 22]. Apicoplast *Pf*TyrRS contains a S4 RNA binding domain whose function remains to be explored [17].

Some aaRSs have been observed to possess *P. falciparum*-specific extensions and insertions that were removed from the recombinant, purified enzymes for structural studies or biochemical characterizations [17, 18, 22, 24–26]. For example, the N-terminal of some aaRSs seems crucial for tRNA binding and the aminoacylation reaction, as suggested for *Pf* aspartyl-tRNA synthetase (AspRS) and *Pf*TrpRS. On the other hand, N-terminal region seems dispensable for the enzymatic activity of *Pf*LysRS, suggesting a regulatory role or non-canonical functionality. The apicoplast copy of *Pf*MetRS contains a unique low complexity 35 amino acid insertion of unknown functionality in the CP1 region [24]. What these extensions are and what is their precise role in the parasite are fascinating issues, which require further study.

## Structures and drug targeting of malarial aaRSs

Theoretically, each aaRS is vital for parasite survival and hence, a potential drug target [6, 37–39]. Over the last half a decade, aaRSs from the malaria parasite have provided many lead inhibitor compounds that can be used to develop species-specific drugs [6, 21, 24, 40–45]. High content screenings have provided aaRS inhibitors as lead anti-malarials [41, 45]. aaRSs are multidomain enzymes and thus provide the flexibility of designing intervention strategies against multiple sites, viz. aminoacylation pocket, editing site, tRNA binding region and additional domains of non-canonical functionalities. Structural studies of malaria parasite aaRSs by X-ray crystallography have hugely boosted the anti-malarial drug discovery programme. Reported anti-malarials that target aaRSs are listed in Table 2 and key targets and their inhibition are discussed below.

**Table 2:** A list of efficient anti-malarial aaRS inhibitors

Inhibitor	Target plasmoDB geneID	Comment
Mupirocin	IleRS PF3D7_1225100	This is a clinical inhibitor of bacterial infection by *S. aeurus*. Likely targets active site of apicoplast IleRS with IC_50_ ~ 90 nM [21]
4-Thiaisoleucine	IleRS PF3D7_1332900	Structural analogue of isoleucine targets the cytoplasmic IleRS [21]
TCMDC-131575	IleRS PF3D7_1332900	Molecule identified in GlaxoSmithKline’s library screening. IleRS is the hypothesized target [45]
Cladosporin	LysRS PF3D7_1350100	A selective malaria inhibitor with IC_50_ value near 50 nM. Kills both liver and blood stage parasites. Drug bound crystal structure is available [40, 41]
Lysyl-adenylate analogues	LysRS PF3D7_1416800	Nearly 50 analogues with μM inihibition reported [46]
Halofuginone	ProRS PF3D7_1213800	Inhibit both liver and blood stages. Bind to parasite enzyme with K_d_ value of 9 nM. Halofuginone bound crystal structure is available [43, 52, 55]
Borrelidin and analogues	ThrRS PF3D7_1126000	Kills *P. falciparum* at IC_50_ near 1 nM. ThrRS inhibition confirmed in enzyme assays [40]. Analogs with reduced cytotoxicity to human were reported [42, 47–49]
A5, A3	AlaRS PF3D7_1367700	Several *P. falciparum* inhibitors identified using in silico screening and docking against active site. A5 was top inhibitor with IC_50_ value near 4 μM [18]
TCMDC-141232	TyrRS PF3D7_1117500	Molecule identified in GlaxoSmithKline’s library screening. Apicoplast copy of TyrRS is the hypothesized target [45]
REP3123 REP8839 C1–C8	MetRS PF3D7_1034900	Known bacterial MetRS inhibitors REP3123 and REP8839 inhibit *P. falciparum* and block translation with IC_50_ values near 150 nM. C1–C8 identified from in silico screening and inhibit parasite growth with IC_50_ values below 500 nM [24]
TCMDC-140014 TCMDC-139627 TCMDC-139450	MetRS PF3D7_1034900	Molecules identified in GlaxoSmithKline’s library screening. MetRS is the hypothesized target [45]
Sulfomyl adenosine analogues	SerRS PF3D7_071770 GluRS PF3D7_1349200 GlnRS PF3D7_1331700 AsnRS PF3D7_0211800 TyrRS PF3D7_0807900	Mechanism based inhibitors that mimic the intermediate aminoacyl-AMP were tested and shown to kill malaria parasite in nM values [42]
AN2729	LeuRS PF3D7_0828200	Member of benzoxaborols family which show anti-malarial activity [42]
TCMDC-140398 TCMDC-140498 TCMDC-140522 TCMDC-140563 TCMDC-140564 TCMDC-140734 TCMDC-141485	PheRS PF3D7_1104000	Molecule identified in GlaxoSmithKline’s library screening. Cytoplasmic copy of PheRS is the hypothesized target [45]

### Targeting single copy aaRSs

Single copy aaRSs, AlaRS, ThrRS, CysRS and GlyRS are important anti-malarial drug targets mainly because targeting of these enzymes would stall translation in three compartments simultaneously [18–20, 29]. Two of these enzymes, AlaRS and ThrRS contain an editing domain, providing an additional advantage to design inhibitors against the editing pocket [18, 19]. In fact, an AlaRS inhibitor A5 has been reported to kill the parasite at low μM values [18]. In an effort to test the known aaRS inhibitors against malaria parasite enzymes, several inhibitors were found to target *P. falciparum* aaRSs [46]. *Pf*ThrRSs was found to be inhibited by the natural compound borrelidin at a remarkable ~ 1 nM IC_50_ value [42]. Borrelidin and its analogs can clear malaria at low concentrations from mice [42, 47]. A major limitation with borrelidin is its lack of specificity for *Pf*ThrRS over the human enzyme, as it’s highly toxic to human cells [42, 47]. Many borrelidin analogues have been synthesized and some of these possess lesser toxicity to human cells and clear malaria both in vitro and in vivo [42, 47–49]. No atomic structures are available for any of these single copy aaRSs, making structural studies of these enzymes for finding anti-malarial drugs a high priority.

### Lysine-tRNA synthetase

**Fig. 4:**
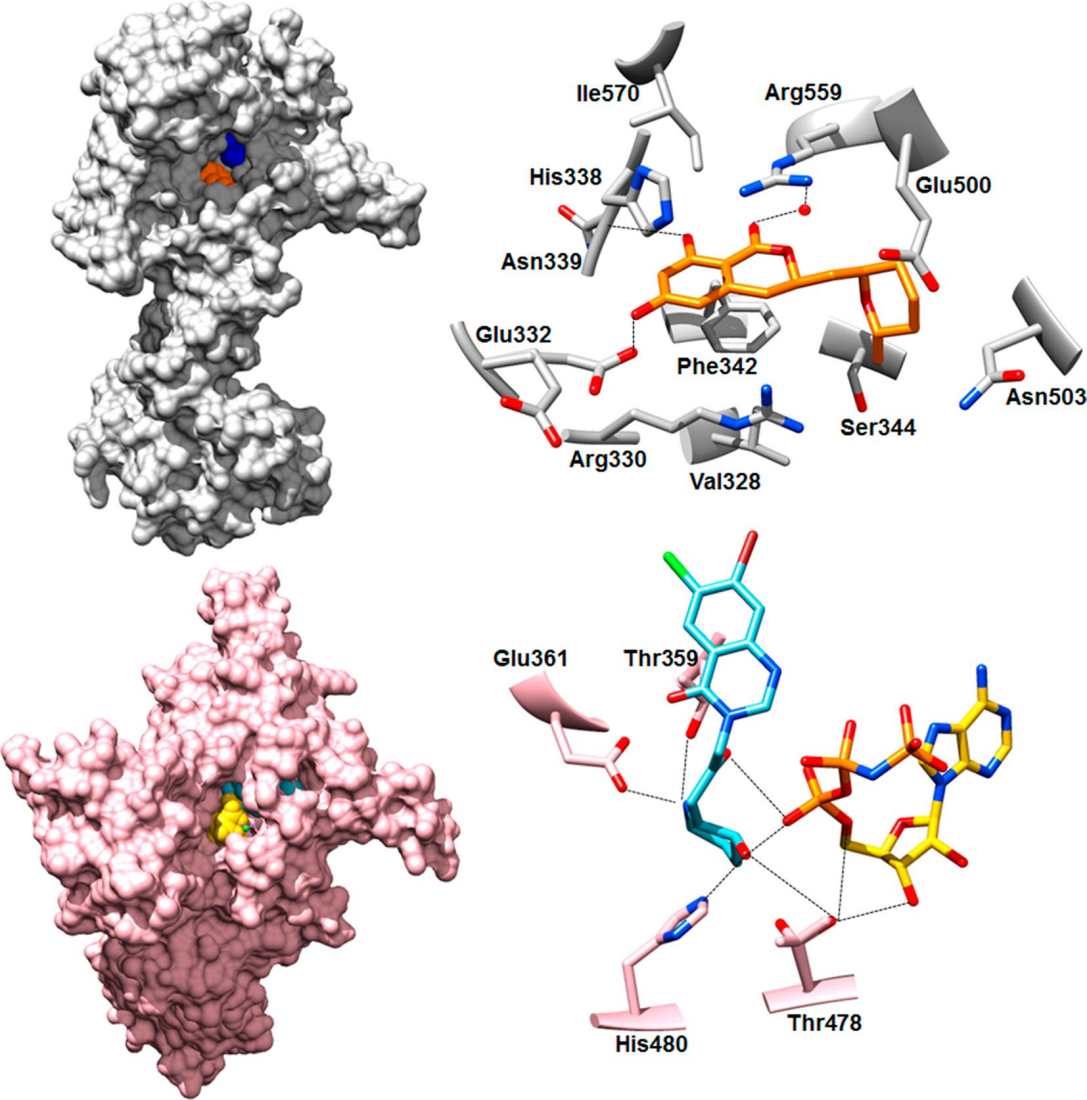
Structures of two drug targets LysRS and ProRS from malaria parasite are shown in drug bound forms. *Upper panel left* shows *Pf*LysRS bound to cladosporin (*orange*) and l-lysine (*blue*). *Upper panel right* shows cladosporin bound to *Pf*LysRS active site. Cladosporin binding is achieved by stacking and hydrogen bonding (shown in *dotted lines*) interactions with the inhibitor. *Red dot* denotes water molecule. *Lower panel left* shows halofuginone (*light blue*) and ATP mimic (*yellow*) bound surface view of ProRS crystal structure. *Lower panel right* shows halofuginone binding in the active site and major interacting residues. Halofuginone biding is stabilized by the ATP binding which makes hydrogen bonds with the inhibitor. Hydrogen bonds are denoted as *dotted lines*

*Pf*LysRS is a class II aaRS and Plasmodium contains two copies of this enzyme; one cytoplasmic and the other apicoplastic [17]. The cytoplasmic copy was reported to be inhibited by a fungal secondary metabolite cladosporin in high content screening (Table 2) [41]. Cladosporin was found to inhibit both blood and liver stages of the parasite with a high specificity over human cells [41, 50]. Structures of both apo and drug-bound forms of *Pf*LysRS have helped in understanding the molecular mechanism of cladosporin binding and specificity over human counterpart [25, 40, 51] (Fig. 4). Cladosporin binds in the adenosine binding site of the enzyme and two main residues-Ser344 and Val328, were proposed to be the specificity regulators [40, 51]. Dissociation constant for *Pf*LysRS with cladosporin was found to be ~ 14 nM, while human LysRS bound the drug at ~ 4 μM [40, 51]. Also, the *P. falciparum* enzyme was observed in a mono-disperse dimeric form whereas the human enzyme was additionally observed in a tetrameric form [25]. The X-ray structure of cladosporin-bound *Pf*LysRS and a detailed dissection of the binding mechanism is expected to assist structure-based drug derivatization of cladosporin. A series of inhibitors were tested against the apicoplastic copy of LysRS and were found to kill the malarial parasite effectively [46].

### Proline-tRNA synthetase

Febrifugine, a Chinese herb derived molecule, and its analogs, especially halofuginone, are highly efficient inhibitors of malaria parasite growth [44, 52–54]. Halofuginone targets both the asymptomatic liver stage and the blood stages of Plasmodium parasites [44, 52, 53]. The cytoplasmic copy of ProRS from malaria parasite was identified as the specific target for these molecules (Table 2) [52, 55]. Crystal structures of malaria parasite ProRS in apo and halofuginone-bound states have revealed the molecular mechanism of inhibition (Fig. 4) [22, 43]. Halofuginone occupies the proline binding pocket and A76 nucleotide at the 3′ end of cognate tRNA [43]. A strong binding of halofuginone was reported to require ATP molecule (K_d_ value of 1 nM) that locks the halofuginone into active site (Fig. 4) [43]. A series of febrifugine and halofuginone analogs have been synthesized by various groups and those functioning as inhibitors in the nanomolar level were tested for their anti-malarial activities in an effort to achieve specificity over the human counterpart [43, 44].

### Tyrosyl-tRNA synthetase

The crystal structure of *Pf*TyrRS was solved at 2.2 Å in complex with tyrosyl-adenylate complex [23]. This structural investigation provided the basis for constitutively active ELR motif in the malarial enzyme. The structure revealed 11 differences in the active sites of human and parasite enzymes, with five in tyrosine binding residues and six involved in AMP binding, which can be used for designing specific inhibitors [23, 45]. In a large scale screening of GlaxoSmithKline’s library, a chemotype potentially targeting the apicoplastic copy of *Pf*TyrRS (TCMDC-141232) was identified (Table 2) [45]. Structural differences in the active site as compared to the human enzyme and its role in a key pathological non-canonical function makes *Pf*TyrRS one of the most attractive drug targets.

### Tryptophanyl-tRNA synthetase

Crystal structures of *Pf*TrpRS have been solved in apo, l-tryptophan-bound and l-tryptophanyl-adenylate-bound forms [26, 56]. These have allowed the exploration of major structural differences between the human and *P. falciparum* enzymes. The ATP binding loop KMSST in the Plasmodium enzyme is present in disordered form, while the ATP binding loop KMSAS of the human enzyme is ordered and in a closed conformation [26]. Cho Yeow Koh et al. suggested a unique targeting strategy against *Pf*TrpRS by focusing on the conformational changes occurring during transition from apo to ligand-bound form rather than only on the active site residues [56]. Similarly, the unique AlaX domain appended to the N-terminus of several aaRSs of Plasmodium parasites can also be targeted.

### Methionyl-tRNA synthetase

Many specific inhibitors targeting the cytoplasmic *Pf*MetRS enzyme have been reported. In the GlaxoSmithKline library screening, four potent inhibitors belonging to two chemotypes, for example, TCMDC-139627 were identified [45]. In another attempt, known MetRS inhibitors REP3123, REP8839 and novel molecules from in silico screening named C1–C8 were found to target malaria parasite growth (Table 2) [24]. Determining the atomic structure of MetRS from Plasmodium would be helpful in understanding the mechanism of inhibition and developing these lead inhibitors into a drug.

### Phenylalanyl-tRNA synthetase

The malaria parasite contains three PheRS proteins; one for each of the three translational compartments [17, 29]. PheRSs show heterogeneity in their functionality and architecture. The cytoplasmic enzyme is an (αβ)_2_ heterotetramer while the mitochondrial and apicoplastic PheRSs are monomeric [29]. GlaxoSmithKline’s library screening identified seven inhibitors belonging to three chemotypes that can target the catalytic α subunit of malarial cytoplasmic PheRS [45]. Structural information for any of the three plasmodial PheRSs is much needed. The presence of three PheRSs in malaria parasite presents an opportunity to block translation in all three compartments.

### Isoleucyl-tRNA synthetase

*Plasmodium falciparum* contains two copies of isoleucyl-tRNA synthetase (IleRS) where one is cytoplasmic and the other one is apicoplastic [18, 21]. Mupirocin is a natural product that selectively targets bacterial IleRS and is the only commercially available antibiotic against aaRSs. Mupirocin was found to target the apicoplastic copy of IleRS at low nano-molar values [21]. GlaxoSmithKline’s library screening has also identified one inhibitor, TCMDC-131575 against *P. falciparum* cytoplasmic IleRS [45]. Interestingly, both copies of parasite IleRS contain editing domains, which provide extra set of pockets to target [18].

## Conclusions

As clear from the above report, malaria parasite aaRSs are not only intriguing for fundamental research, but are also validated drug targets. The apicoplast and mitochondrial translational setups are equally druggable as the cytoplasmic counterpart, and require more studies targeted at exploring their structures and mechanisms. Available inhibitors of bacterial-type organellar aaRSs suggest that their targeting is feasible. Many of the cytoplasmic aaRSs remain to be explored for their structure and physiological roles. Previous studies have hinted at parasite specific adaptations in housekeeping aaRS enzymes, making the predicted extra domains in non-characterized aaRSs, fascinating to study. Moreover, aaRSs are conserved enzymes and thus repurposing of drugs developed against malarial aaRSs can be used to target other eukaryotic pathogens and hence be of much value.

## Abbreviations

aaRS: aminoacyl-tRNA synthetase
AlaX: alanine-tRNA synthetase editing domain homolog
MetRS: methionyl-tRNA synthetase
TyrRS: tyrosyl-tRNA synthetase
